# Loss of Heterozygosity and Copy Number Alterations in Flow-Sorted Bulky Cervical Cancer

**DOI:** 10.1371/journal.pone.0067414

**Published:** 2013-07-09

**Authors:** Sabrina A. H. M. van den Tillaart, Wim E. Corver, Dina Ruano Neto, Natalja T. ter Haar, Jelle J. Goeman, J. Baptist M. Z Trimbos, Gertjan J. Fleuren, Jan Oosting

**Affiliations:** 1 Leiden University Medical Center, Department of Gynecology, Leiden, The Netherlands; 2 Leiden University Medical Center, Department of Pathology, Leiden, The Netherlands; 3 Leiden University Medical Center, Department of Medical Statistics, Leiden, The Netherlands; Sapporo Medical University, Japan

## Abstract

Treatment choices for cervical cancer are primarily based on clinical FIGO stage and the post-operative evaluation of prognostic parameters including tumor diameter, parametrial and lymph node involvement, vaso-invasion, infiltration depth, and histological type. The aim of this study was to evaluate genomic changes in bulky cervical tumors and their relation to clinical parameters, using single nucleotide polymorphism (SNP)-analysis.

Flow-sorted tumor cells and patient-matched normal cells were extracted from 81 bulky cervical tumors. DNA-index (DI) measurement and whole genome SNP-analysis were performed. Data were analyzed to detect copy number alterations (CNA) and allelic balance state: balanced, imbalanced or pure LOH, and their relation to clinical parameters.

The DI varied from 0.92–2.56. Pure LOH was found in ≥40% of samples on chromosome-arms 3p, 4p, 6p, 6q, and 11q, CN gains in >20% on 1q, 3q, 5p, 8q, and 20q, and losses on 2q, 3p, 4p, 11q, and 13q. Over 40% showed gain on 3q. The only significant differences were found between histological types (squamous, adeno and adenosquamous) in the lesser allele intensity ratio (LAIR) (p = 0.035) and in the CNA analysis (p = 0.011). More losses were found on chromosome-arm 2q (FDR = 0.004) in squamous tumors and more gains on 7p, 7q, and 9p in adenosquamous tumors (FDR = 0.006, FDR = 0.004, and FDR = 0.029).

Whole genome analysis of bulky cervical cancer shows widespread changes in allelic balance and CN. The overall genetic changes and CNA on specific chromosome-arms differed between histological types. No relation was found with the clinical parameters that currently dictate treatment choice.

## Introduction

### Prognostic factors for cervical cancer

Cervical cancer is one of the most frequent gynecological cancers worldwide. Following the surgical treatment of cervical tumors, prognostic factors for survival include the clinical parameters FIGO stage, tumor diameter, tumor in the parametria, tumor positive pelvic lymph nodes, vaso-invasion, and infiltration depth. Histological type is also related to prognosis, and is evaluated both pre- and postoperatively [1]–[4]. Although parameters can be partly determined pre-operatively by clinical examination, imaging, or the pathological evaluation of biopsy specimens, most parameters are only definitively established following the post-operative pathological examination of surgical specimens. Presence or absence of these factors is of prognostic relevance and is therefore used to select both the primary treatment, and to decide whether adjuvant chemotherapy and/or radiotherapy are necessary.

Surgical treatment is considered to be the optimal primary treatment for small diameter cervical tumors (<4 cm, FIGO stage <1b2). Locally extended tumors (FIGO 2b or higher) are primarily treated by chemo-radiation. There is, however, no worldwide agreement on the optimal primary treatment for bulky cervical cancer (diameter >4 cm, FIGO ≥1b2–2b), although radiotherapy or surgery are options [5]–[13]. Recently, our group reported a possible additional prognostic factor for bulky cervical tumors. Patients with barrel-shaped (lateral extension ≥1.5× craniocaudal extension) bulky tumors showed a worse disease-free and overall survival after surgical treatment, when compared to exophytic (all other) tumors. Primary surgical treatment, rather than radiotherapy or chemo-radiation, has been proposed as the optimal treatment for patients with exophytic bulky tumors [14].

The ability to select more homogenous subgroups of patients with cervical tumors may help in the selection of the most suitable treatment strategy for individual patients. Identification of patients with specific genetic patterns might be a way to achieve this goal. Genetic changes could be objectively assessed, pre-operatively, in tumor biopsies, potentially providing a more accurate prediction of stage and clinical behavior than the physical examination of the patient. Furthermore, genetic profiling could provide information on the genes or pathways responsible for tumor growth and metastasis.

### Genetic profiling

The progression of normal cells to cancer is accompanied by changes in DNA, and genetic profiles have been established for several types of cancer. These profiles have been largely determined using arrayCGH, and have therefore been limited to copy number changes. In this study, we used single nucleotide polymorphism (SNP) arrays to determine the genetic profile of flow-sorted tumor populations. This approach has the advantage of also determining allele-specific changes, in addition to copy number alterations (CNA), in pure tumor cells. In order to include loss of heterozygosity (LOH) in the analysis, we developed the lesser allele intensity ratio (LAIR) approach, which allows the assessment of discrete allele specific copy numbers (CN) for all genomic locations [15]. This method allows the classification of the discrete total CN as both the sum of two alleles and as the balance state, which can then be divided into 3 classes: balanced, imbalance, and LOH.

The statistical analysis of differences in genetic profiles between groups of tumors has proven to be difficult. The nature of the genetic changes in tumors causes strong correlations between measurements from neighboring probes, correlations that are not properly handled in commonly used statistical tests. In this study we introduce a statistical method based on the global test [16], which performs multiple testing correction correctly in the presence of strongly correlated values. Another advantage of the global test is that it can test the hypothesis that groups of samples are the same on a whole genome level, and can zoom in on chromosome arms when a difference between groups is found.

### Aim of the study

The purpose of this study was to identify genetic changes associated with one or more prognostic factors in cervical cancer patients. Our approach was to use SNP array analysis on flow-sorted FFPE tumor tissue from bulky cervical cancers.

To our knowledge, this is the first large scale, whole genome SNP array study of this stage of cervical tumors, and no publication has yet described a genomic profile of cervical tumors based on the SNP array analysis of pure tumor tissue. Additionally, this is the first whole genome SNP array study of a large group of bulky cervical tumors in relation to genetic changes in balance state and CN, and their relationship to unfavorable prognostic factors.

## Materials and Methods

### Samples

Tissue from 107 cervical carcinomas, as well as paired normal (non-affected) endometrial and/or lymph node tissue, was obtained from the FFPE tissue bank of the Department of Pathology, Leiden University Medical Center (LUMC). Samples were handled in accordance with the medical ethical guidelines described in the Code Proper Secondary Use of Human Tissue established by the Dutch Federation of Medical Sciences (www.federa.org). Our study group consisted of patients living in the Netherlands and in Suriname. All patients who presented with bulky cervical cancer FIGO stage ≥1b2–2b and received primary surgical treatment at the LUMC between January 1984 and November 2000, were included in the study group. A large number of clinical parameters have been characterized in these patients, but for this study we choose to investigate seven clinical parameters known to be of prognostic value: tumor diameter, histological type, parametrial involvement, pelvic lymph node status, vaso-invasion, infiltration depth, and growth pattern. The growth pattern was defined as barrel-shaped if the lateral extension of the tumor was ≥1.5× the craniocaudal extension of the tumor; otherwise the tumor was classified as exophytic. Histological typing (squamous, adeno, adenosquamous, or mixed tumors) was based on histochemical staining with H&E, periodic acid-Schiff (PAS) reagent, and Alcian blue for mucin detection. The use of this additional staining is well established among Pathologists [17]. FIGO stage was not included in the analyses since the various postoperative characteristics are overlapping with, and more accurate than the characteristics that are used for the pre-operative FIGO staging.

### Tissue sample preparation

Paraffin sections taken from all samples were H&E stained and reviewed by a pathologist (GJF). The tumor nodule was marked on the H&E section, which was used as a guide to trim the normal tissue from paraffin block prior to flow cytometric workup. The tumor negative status of blocks containing normal tissue (either tumor negative lymph nodes or endometrial tissue) was reviewed by histology and confirmed. Cell suspensions were prepared for flow cytometry, as described in detail elsewhere [18], [19]. Briefly, six to ten 60 μm sections were taken from each paraffin block. Sections were dewaxed and further processed until a cell suspension was obtained. Cells were then harvested, washed, counted and stored on ice prior to further processing.

### Immunocytochemistry of cell suspensions

Immunocytochemistry has been described in detail elsewhere [18], [19]. Briefly, five million cells were incubated in a mixture of monoclonal antibodies directed against keratin or vimentin. The following MAbs were used: anti-keratin MNF116 (DAKO, Glostrup, Denmark), anti-keratin AE1/AE3 (Millipore-Chemicon, Billerica, MA), and anti-vimentin V9-2b (diluted 1∶5) (Antibodies for Research Applications BV, Gouda, The Netherlands). Cells were incubated with premixed FITC- or RPE-labeled secondary reagents (Goat F(ab2)' anti-mouse IgG1-FITC and goat F(ab2) ' anti-mouse IgG2b-RPE [Southern Biotechnology Associates, Birmingham, AL]), and DNA was labeled with DAPI (Sigma-Aldrich, Zwijndrecht, Netherlands) [20].

### Flow cytometry and sorting

Using an LSRII (BD Biosciences, Erembodegem, Belgium) flow cytometer, a gate was created to collect 20,000 keratin-positive single cell events during acquisition. Standard filter sets were used for the detection of FITC, R-PE and DAPI fluorescence. A data file contained all events. The WinList 6.0 and ModFit 3.2.1 software packages (Verity Software House, Inc., Topsham, ME) were used for data analysis and DNA index (DI) calculation (median of G_0_G_1_ population of tumor cell fraction / median of G_0_G_1_ population of stromal cell fraction). In order to separate tumor cells from genetically normal cells (connective tissue, lymphocyes and blood vessels) [21], [22]. keratin-positive and vimentin-positive normal cells were collected separately, using a FACSAria I flow-sorter at 40 psi (BD Biosciences, Erembodegem, Belgium). In cases where flow cytometry detected more than one population of keratin-positive tumor cells, both populations were sorted independently. The most prevalent DNA population was selected to undergo SNP array analysis.

DNA isolation was performed as previously described [23]. In cases where endometrial or lymph node tissue was not available, DNA from sorted tumor stroma cell fractions was used as a reference [23].

### SNP array

**Figure 1:**
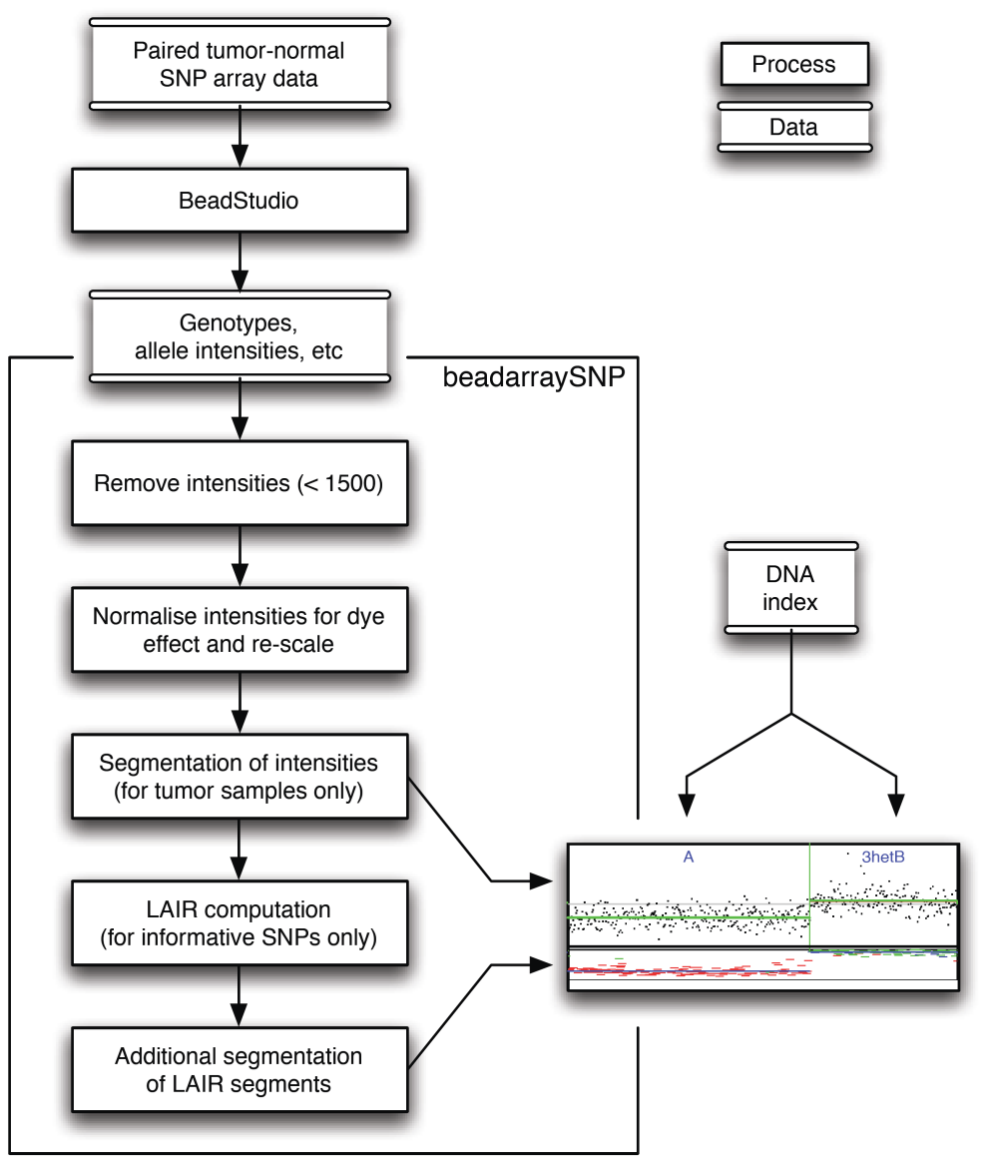
Analysis steps for SNP array data with the Illumina Beadstudio and the beadarraySNP package. The data is preprocessed to obtain quality checked and normalized values for intensity and LAIR. Together with the DNA-index these are interpreted on a discrete and categorical scale. The embedded plot shows normalized intensity as dots in the upper panel, and LAIR in the lower panel as hyphens. The segmentation procedure has divided this region in two segments. The left segment has copy number 1 with LOH. The allelic state is A. The right segment has copy number 2 with balance. The allelic state is AB.

The Golden gate Linkage panel V, consisting of 4 arrays with a total of 6000 SNPs (Illumina, San Diego, USA), was used to analyze tumor-derived DNA, together with DNA from matched normal/non-affected tissue. Assays were performed as previously described [24]. The resolution of this assay is relatively low, but in contrast with other SNP assays it can be used with FFPE derived DNA [24], [25]. These SNP analyses on FFPE tissue were extensively validated previously by FISH analysis [15], [24]–[26]. Samples were processed in 6 batches of 48 samples, and samples of the same patient were always processed in the same batch. A 7^th^ batch was used to repeat assays with low quality. The samples were genotyped in Illumina Beadstudio 2.3. The reference genotype clusters were derived from the normal samples in the dataset, and genotypes and allele intensities were extracted. The beadarraySNP package was used for further data processing. The analysis steps are depicted in Figure 1. SNPs that deviated significantly from Hardy-Weinberg equilibrium (at a significance level of 0.05, divided by the number of SNPs analyzed  = 0.00001), and SNPs with a call rate lower than 95% in controls, were removed to prevent the possibility of genotyping errors. All assays with a median intensity of below 2000 for one of the alleles were rejected and repeated in batch 7. Normalization was subdivided in 4 steps: I) normalize intensities for dye effect – quantile normalization was applied to make the distribution of the intensities for both dyes identical, II) per sample between assay quantile normalization to equalize intensity differences, III) within sample normalization to scale the median intensity of each allele to 1, IV) per SNP between sample normalization using the reference samples (which scales the total intensity of SNPs and corrects the allele specific bias by using a linear model between the B-allele ratio and total intensity). Matched normal samples were used to select the informative heterozygous SNPs. The LAIR was calculated for all informative SNPs in the tumor samples. The LAIR value is basically the B-allele ratio, the ratio of the intensity of the B-allele and the total intensity, mirrored on its symmetry axis at 0.5, and scaled to a value between 0 and 1. This makes it easy to compute averages and enables segmentation. In order to identify genomic regions with identical CN and balance state, the data were segmented using circular binary segmentation at the default settings of the DNAcopy R package [27]. For each tumor, first the signal intensity of each chromosome was segmented, followed by a sub-segmentation of the previously obtained segments of the LAIR [15]. By combining the continuous CN (signal intensity) and the LAIR value of a segment with the sample DNA index measured by flow cytometry, we developed a new calling method that assigns an allelic state to each segment [15]. In the present study allelic state was distinguished along 2 dimensions. For each segment, discrete CN and a balance state was assigned. The discrete CN is an absolute measure assigned to segments in such a way that the average discrete CN values across all segments should reflect the sample DNA index. The balance between alleles was divided in 3 possible outcomes: balanced segments – with the same CN for both alleles and a LAIR value near 1; LOH – segments with only one allele present and a LAIR value near 0; imbalanced segments – with different CN for the two alleles and a LAIR value between 0 and 1.

### Statistical analysis

The breakpoints between segments are different in every sample. The breakpoints of each sample were applied to all samples. This makes the samples comparable after segmentation The global test was used to detect differences in genetic changes between groups of patients at the whole genome level and in chromosomal arms [16]. We tested differences both in continuous and discrete CN. The continuous CN is a relative measure; the sample average is 1 regardless of the DI. Discrete CN is an absolute measure that represents the number of copies in a genomic segment in each cell of a tumor. The continuous CN is the conventional way of looking at CN; we would investigate here whether it is useful to look at absolute CN. LAIR as a continuous measure of allelic balance and balance state as a discrete measure were also tested. Continuous CN gains and losses were defined as deviating more than 15% from the sample average. The global test allows the use of confounders. Ethnic group was used as a confounder for all tests, because the genetic make up could influence the occurrence of chromosomal changes. DI was used as an extra confounder in the analysis of continuous CN, because DI has a relationship with gains and losses. The determination of discrete CN already takes the DI into account, and therefore using DI as a confounder would render this analysis back to a relative measure instead of an absolute (see Figure S2 in File S1). Ethnicity was defined by clustering the genotypes together with HapMap samples of known ethnic origin. The tests were further localized by performing the global test on all chromosomal arms individually. Differences between groups were accepted as significant when the false discovery rate (FDR) was lower than 0.05 [28].

All of our SNP-data can be found in the Gene Expression Omnibus: series GSE29143.

## Results

### Clinical data

From the 107 cervix carcinoma patients included in the study, sufficient DNA material was obtained for 82 matched tumor/normal pairs after flow sorting. One sample was removed after hybridization due to low data quality. Table 1 shows summarized clinical information on the 81 patients analyzed, while Table S1 in File S1 shows the data for individual patients. As 96.3% of the tumors were found to have a tumor diameter larger than 40 mm, this parameter was not explored further.

**Table 1:** Clinical data of 81 SNP analyzed patients.

		Mean	Min – Max
Age	(yrs)	47	25–87
		N	%
**Place of residence**	Netherlands	47	58
	Surinam	32	39.5
	Other	2	2.5
**Ethnic cluster**	European	45	55.6
	African	25	30.9
	Asian	11	13.6
**FIGO stage**	1b2	59	72.8
	2a	19	23.5
	2b	3	3.7
**Histological type**	squamous carcinoma	52	64.2
	Adenocarcinoma	3	3.7
	adenosquamous carcinoma	20	24.7
	other/mixed	6	7.4
**Growth pattern**	Exophytic	40	49.4
	barrel shaped	41	50.6
**Infiltration depth**	0–5 mm	4	4.9
	6–10 mm	11	13.6
	11–15 mm	16	19.8
	>15 mm	44	54.3
**Tumor diameter**	21–40 mm	1	1.2
	>40 mm	78	96.3
	Unknown	2	2.5
**Tumor in parametria**	No	57	70.4
	Yes	24	29.6
**Tumor in lymphnodes**	No	47	58
	Yes	34	42
**Vaso-invasion**	No	36	44.4
	Yes	39	48.1
	Unknown	6	7.4

Principal Components Analysis (PCA) was performed using the 4 original HapMap populations as reference panels, together with genotypes obtained from the patients' normal tissue. Three major genetic clusters could be distinguished in the four HapMap populations, with the Japanese and Chinese HapMap populations clustering together. Guided by this clustering, we classified the patients into 3 ethnic groups: European (EUR, n = 45) for patients that cluster together with the CEU HapMap population, African (AFR, n = 25) that cluster together with YRI, and Asian (ASI, n = 11) that cluster together with the CHB and JPT populations. For more detailed information, see Figure S1 in File S1.

### Samples

**Figure 2:**
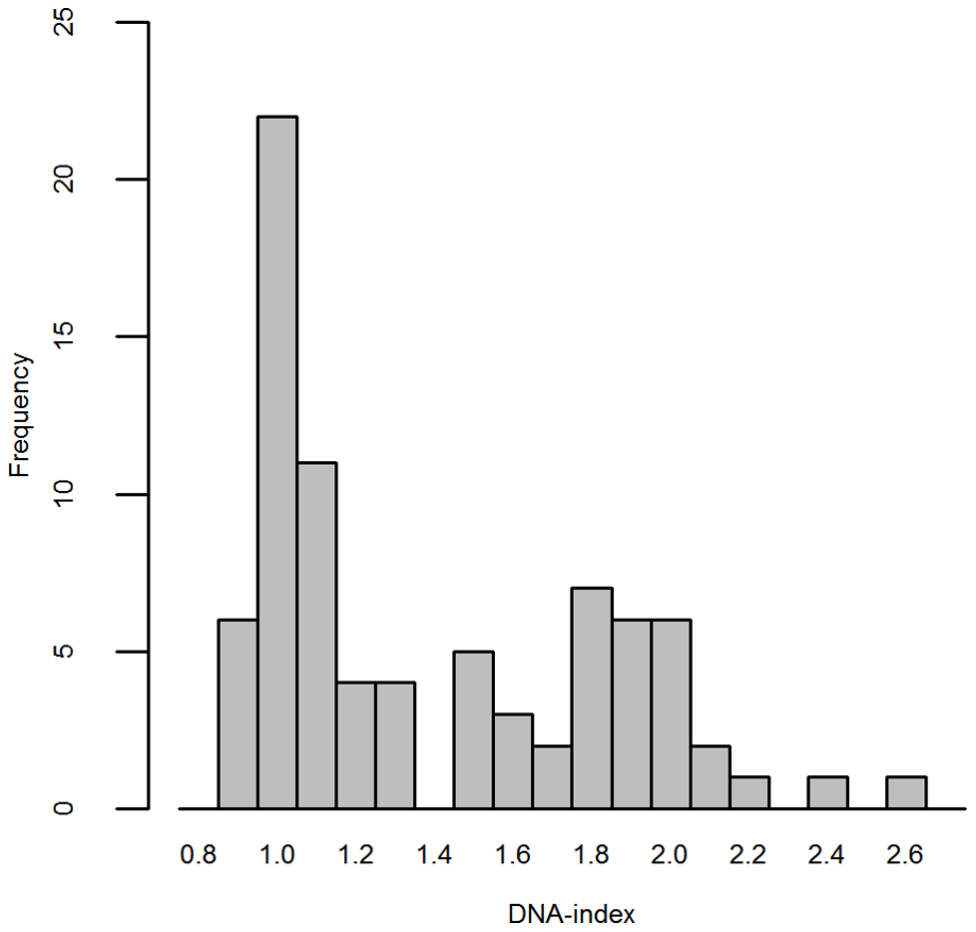
Distribution of the DNA index in 81 cervical tumor samples. There is a bimodal distribution of the DNA index with peaks around 1, and close to 2.

Most of the 81 matched tumor samples (89%) could be paired with normal/non-affected tissue. As normal tissue was not available in 9 cases, the tumor DNA was instead paired with the DNA from normal stroma cells obtained from the flow sorting procedure [23]. Cell sorting detected the presence of more than one tumor cell population in 8 cases. For these cases, the most prevalent DNA population was selected to undergo SNP array analysis. The DNA-index (DI) of flow-sorted samples varied from 0.92 to 2.56. Figure 2 shows the DI density plot of the patient group.

### Overall genetic pattern

#### Balance State

**Figure 3:**
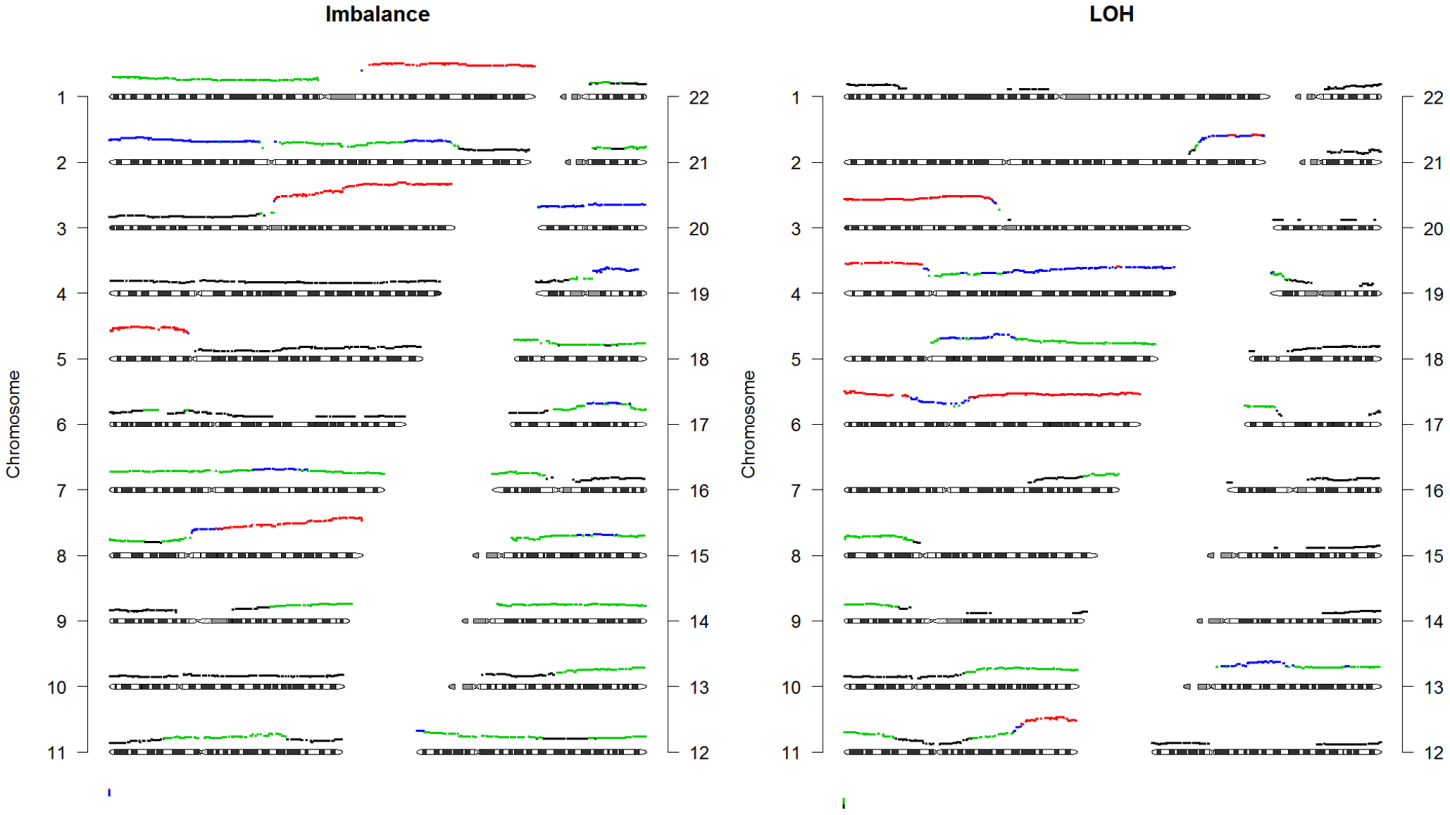
Overall frequency of imbalance and LOH per chromosome. Besides the height of the graph, The colors indicate the frequency of LOH; black: >10%, green: >20%, blue: >0%, red: >40%. The balanced group is not plotted; it is the complement of these 2 groups.

When looking at the balance state patterns generated by the analysis of the SNP array data, it can be observed that LOH is present in almost all chromosomal regions (Figure 3). On 28 out of 40 chromosome arms, more than 10% of the patients showed LOH. LOH was particularly frequent on chromosome arms 3p, 4p, 6p, 6q, and 11q, where it was observed in more than 40% of all patients. Besides LOH all chromosomal regions show the presence of imbalances in at least 10% of the samples (Figure 3). The pattern of imbalance is somewhat complementary to the pattern of LOH.

#### Copy number alterations

**Figure 4:**
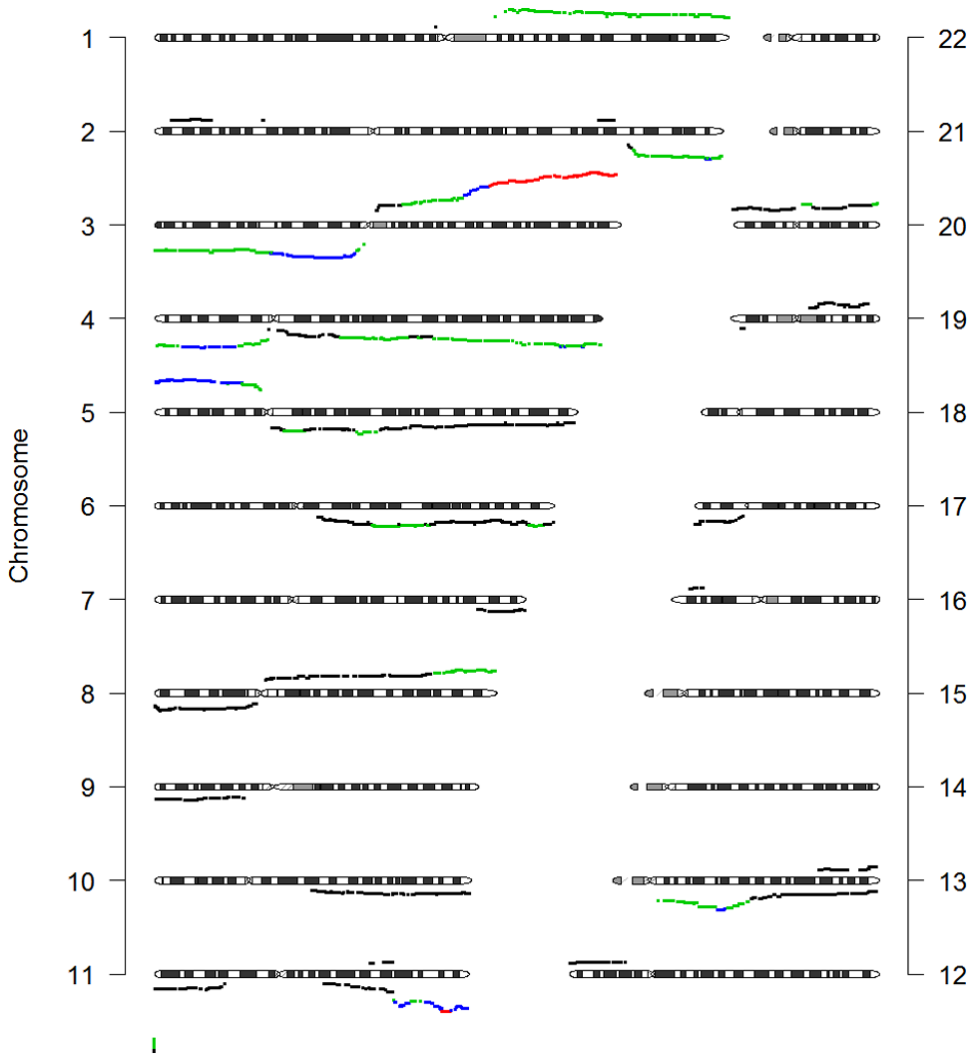
Frequency of gains and losses. Gains are depicted on top of the ideograms, while losses are depicted below. The colors indicate the frequency within the dataset; black: >10%, green: >20%, blue: >30%, red: >40%. Gains and losses were identified when the continuous CN deviated more than 15% from the sample average.

CNA using the continuous CN can be seen throughout the genome (Figure 4). More than 20% of the patients show gains on 1q, 3q, 5p, 8q, and 20q and losses on chromosomes 2q, 3p, 4p, 11q, and 13q. Gain on 3q was found in >40% of all samples.

### Relation between clinical parameters and genetic changes

#### Balance state

Table 2A shows the results of the whole genome analysis of LAIR and the balance state. When the 22 autosomes and the X chromosome were analyzed together, only histological type showed statistically significant differences in LAIR value (p = 0.035). No differences between the different clinical parameters were observed in balance state (p = 0.050). Focusing on the chromosome arms individually showed that the difference between histological groups could not be attributed to a specific chromosome arm.

**Table 2:** Whole genome analyses of genetic changes and clinical parameters.

A. Whole genome analysis of balance *		
	LAIR	Allelic Balance
Clinical parameter	*p-value*	*p-value*
Growth pattern	0.244	0.184
**Histological type**	**0.035**	0.05
Infiltration depth	0.651	0.509
Lymphnodes	0.626	0.683
Parametria	0.928	0.769
Vasoinvasion	0.146	0.301

B. Whole genome analysis of Copy Number *			
	Continuous variables	Discrete variables	
			extra confounder DI
Clinical parameter	*p-value*	*p-value*	*p-value*
Growth pattern	0.346	0.209	0.396
**Histological type**	**0.011**	0.363	**0.019**
Infiltration depth	0.397	0.967	0.338
Lymphnodes	0.614	**0.032**	0.637
Parametria	0.695	0.383	0.654
Vasoinvasion	0.213	0.744	0.083

* *confounder: Ethnic cluster*.

#### Copy number alterations

**Figure 5:**
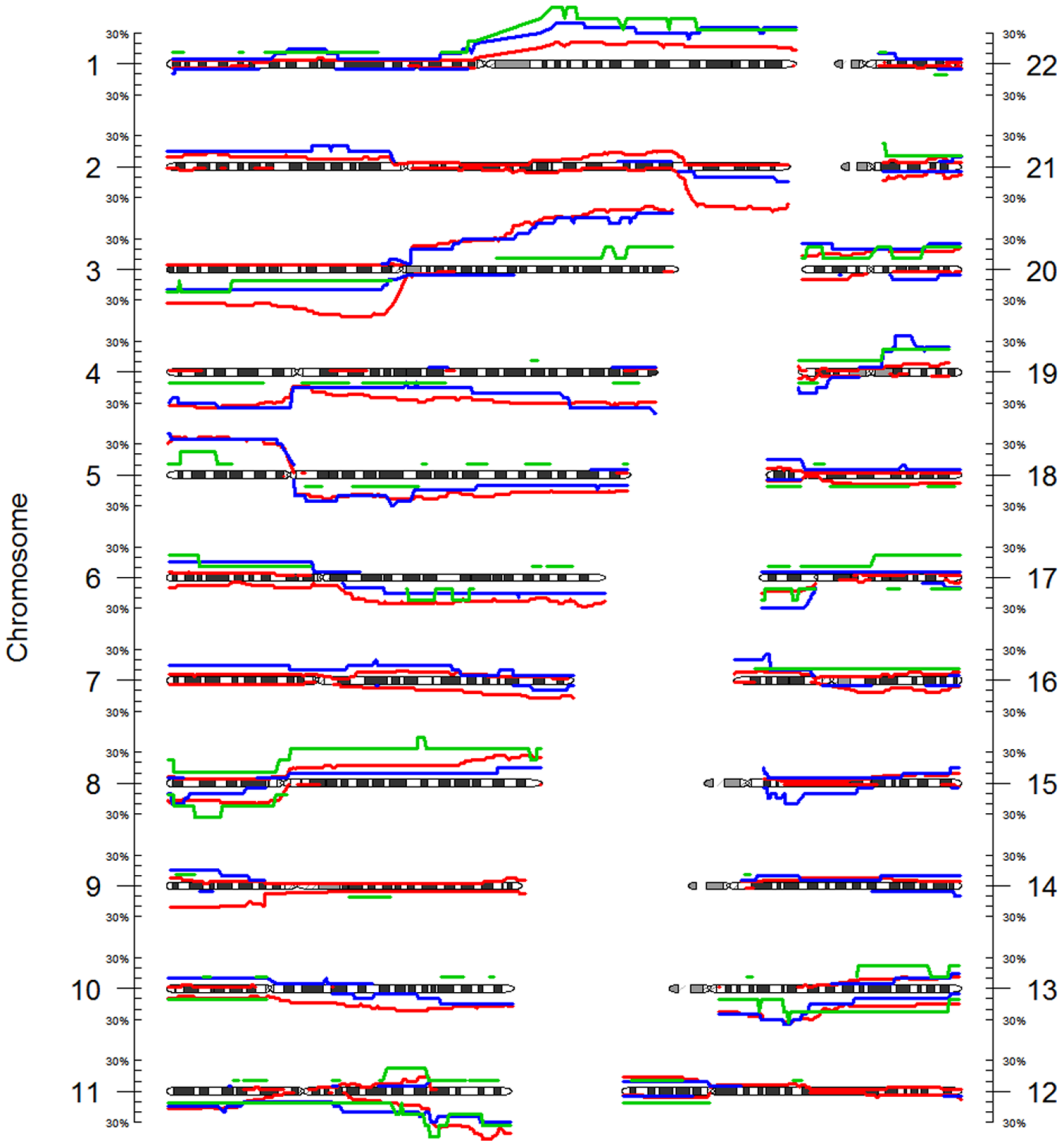
Frequency of gains and losses in histological groups. Gains are depicted on top of the ideograms, while losses are depicted below. Red: squamous tumors, green: adenocarcinoma + mixed type, blue: adenosquamous tumors. Adenocarcinoma and mixed type were combined because of the low numbers of samples in these groups.

Table 2B shows the result of the whole genome analysis for changes in CN. Continuous CN values showed statistically significant differences only for histological types (p = 0.011). The test for discrete CN was not statistically significant (p = 0.363). When DI is added as a confounder to the analysis of discrete CN the difference between histological types is significant (p = 0.019), The discrete CN profiles of patients with and without lymph node metastasis were significantly different (p = 0.032), and here the test for continuous CN was not significant (p = 0.614). The inclusion of DI as a confounder in the test for discrete CN now shows no difference anymore (p = 0.637). See also Figure S3 in File S1. For continuous CN the tests show comparable results with or without inclusion of DI as a confounder (data not shown). We then zoomed in on individual chromosome arms when analyzing the clinical parameters that showed a difference. Squamous tumors showed greater losses on 2q (FDR = 0.004), while adenosquamous tumors were found to have more gains on 7p, 7q, and 9p (FDR = 0.006, FDR = 0.004, and FDR = 0.029 respectively). For discrete CN, the differences between groups with and without lymph node involvement could not be attributed to any of the chromosome arms in particular. Figure 5 shows the differences in continuous CN for histological type on the different chromosome arms.

## Discussion

Using SNP array analysis, we have shown extensive genome-wide LOH and CN changes in bulky cervical cancer. To our knowledge, no previous studies have applied genome-wide genetic profiling to such a large number of bulky cervical cancers (FIGO stage 1b2–2b).

The analysis of the relation between genetic changes and the prognostic factors histological type, infiltration depth, lymph node status, extension to the parametria, vaso-invasion, and growth pattern revealed a statistically significant difference in the analysis of the discrete CN between groups with and without lymph node involvement, and a relationship between histological type and changes in LAIR and continuous CN. Even though the discrete CN did not show differences between the different histological types, we observed that when the DNA index was used as a confounder in the analysis the significant difference between the histological groups was restored (p-value goes from 0.363 to 0.019). This signifies that the discrete CN values contain ample information to distinguish the groups, but that relative changes in DNA compared to the average DNA content of a cell are more important for the difference between histological groups than the absolute allele count.

In order to specify the genetic changes that contribute to the differences in clinical parameters, we analyzed chromosome arms individually. In the analysis of continuous CN changes, the histological group squamous carcinomas showed a statistically significant increase in loss on 2q, while adenosquamous carcinomas showed more gains on 7p, 7q, and 9p. The statistical differences in these 4 regions are comparable, but the numerical difference is most pronounced for chromosome 2q, where around 10% of the adenosquamous carcinomas shows loss, but over 40% of the squamous carcinomas shows loss. Despite the whole genome difference in discrete CN between patients with and without lymph node involvement, we could not link this difference to a specific chromosomal arm. Thus, this difference cannot be used to extract a clinical parameter.

CNA were found in previous studies using classical array-CGH, which examines only continuous CN changes (see Table S2 in File S1) [29]–[39]. As can be seen in the Table, the gains that we found were previously reported:

Gains on 1q were also found in 8 of the 11 included studies, on 3q frequently in all of the studies, on 5p in 5 of the 11 studies, on 8q in 3 of the other studies, and on 20q in 6 studies. The losses on 2q were reported in 5 of the 11 other studies, on 3p in 8 of 11, on 4p in 6, on 11q in 6, and on 13q in 8 of the other studies. All of our findings were previously found by Rao et al., and all but one by Lando et al., although additional changes on other locations were also found in these studies. This might be explained by the inclusion of tumors with a higher FIGO stage in these studies.

Some of the studies also investigated the relation between clinical parameters and genetic changes in cervical cancer (see Table S2 in File S1) [29], [30], [33], [34], [36]. Rao et al. found no differences between squamous and adeno tumors stage 1b–4b. This may be due to the small number of adenocarcinomas included (5 adenocarcinomas and 72 squamous carcinomas) [34]. There were no adenosquamous tumors in this group. In an analysis of stage 1b–3b cervical tumors, Wilting et al. reported significantly more gains in 9 squamous tumors as compared to 7 adenocarcinomas. Higher gains were predominantly found on 3q [36], although the method used to define the histological type was not described. The locations of the differences in genetic alterations between squamous and adenosquamous carcinomas in our study did not coincide with the findings of Wilting et al., but there were no adenosquamous tumors in this group.

The difference in overall findings between our and previous studies may be explained by differences in FIGO stage, sample size, and staining techniques to discriminate histological type. The group of tumors that we used was not previously analysed in the literature, and, except for lymph nodes, the clinical parameters that we analysed were studied before in only 2 of the other 11 studies (Rao et al., and Wilting et al.). Dilution caused by the use of tumor tissue instead of pure tumor cells may also explain differences in results.

The prognostic relevance of histological type is still an object of debate and is not currently used for primary or adjuvant treatment choice [1]–[4], [40]–[42]. The conflicting results of these studies on the effect of histological type on tumor behavior and patient survival may also be due to differences in the classification of tumors, where no specific staining (PAS and Alcian blue) was applied. Future studies on genomic alterations are advised to take the different genetic profiles of histological types into consideration.

The regions with the most prominent differences contain many genes. The aim of this study was to find additional pre-operative parameters that could be used for treatment choice, without knowing the causative gene. This topic deserves further investigation.

The SNP array used in this study consisted of 6000 SNP markers distributed evenly over the genome, and the array was optimized to have a higher minor-allele frequency in the Caucasian (European) population. As a consequence, the average number of informative probes was higher for the Caucasian patients (2139 informative SNPs) than for the African (1796) and Asian (1712) patients. This may result in an underestimation of the genetic changes that take place in cervical cancer in these populations. Although it would have been interesting to compare genetic changes and the relationship to clinical parameters by ethnic background, the subgroups are too small to allow this.

Our results revealed genetic changes related to histological type, a clinical parameter not currently used for treatment choice. Our analysis showed no relation of genetic changes to clinical parameters that could be used to predict unfavorable post-operative prognostic characteristics or select subgroups of patients. It seems that, at present, the best evaluation of prognostic factors still comes from pre-, intra-, and post-operative findings of the gynecologic oncologist and pathologist. As differences in DNA alterations between tumors of different histological types may have an impact on tumor behavior, treatment response and survival, future research should include larger patient groups, and use staining techniques that can reliably distinguish histological type.

## Supporting Information

File S1 **Figure S1, Classification of patient ethnicity in relation to HapMap populations.** Red dots are patients, while blue, yellow, green, and pink dots correspond to European, African, Japanese and Chinese populations, respectively. **Figure S2, Balance state classification in relation to continuous CN and LAIR.** Red dots indicate balanced state, blue dots imbalanced state and green dots indicate LOH. **Figure S3, Relationship between discrete and continuous CN.** The blue dots represent the continuous CN, while the orange dots represent continuous CN multiplied by sample DI. **Table S1, Clinical details for individual samples. Table S2, Main characteristics and results of array-CGH studies on cervical tumors (27–37).**
(DOC)
