# Supplementary material for: Loss of Heterozygosity and Copy Number Alterations in Flow-Sorted Bulky Cervical Cancer
Source: PLoS One. 2013 Jul 9;8(7):e67414. doi: 10.1371/journal.pone.0067414 (PMC3706587; doi:10.1371/journal.pone.0067414)
Supplement: File S1 — Figure S1, Classification of patient ethnicity in relation to HapMap populations. Red dots are patients, while blue, yellow, green, and pink dots correspond to European, African, Japanese and Chinese populations, respectively. Figure S2, Balance state classification in relation to continuous CN and LAIR. Red dots indicate balanced state, blue dots imbalanced state and green dots indicate LOH. Figure S3, Relationship between discrete and continuous CN. The blue dots represent the continuous CN, while the orange dots represent continuous CN multiplied by sample DI. Table S1, Clinical details for individual samples. Table S2, Main characteristics and results of array-CGH studies on cervical tumors (27–37). (DOC) [file pone.0067414.s001.doc]

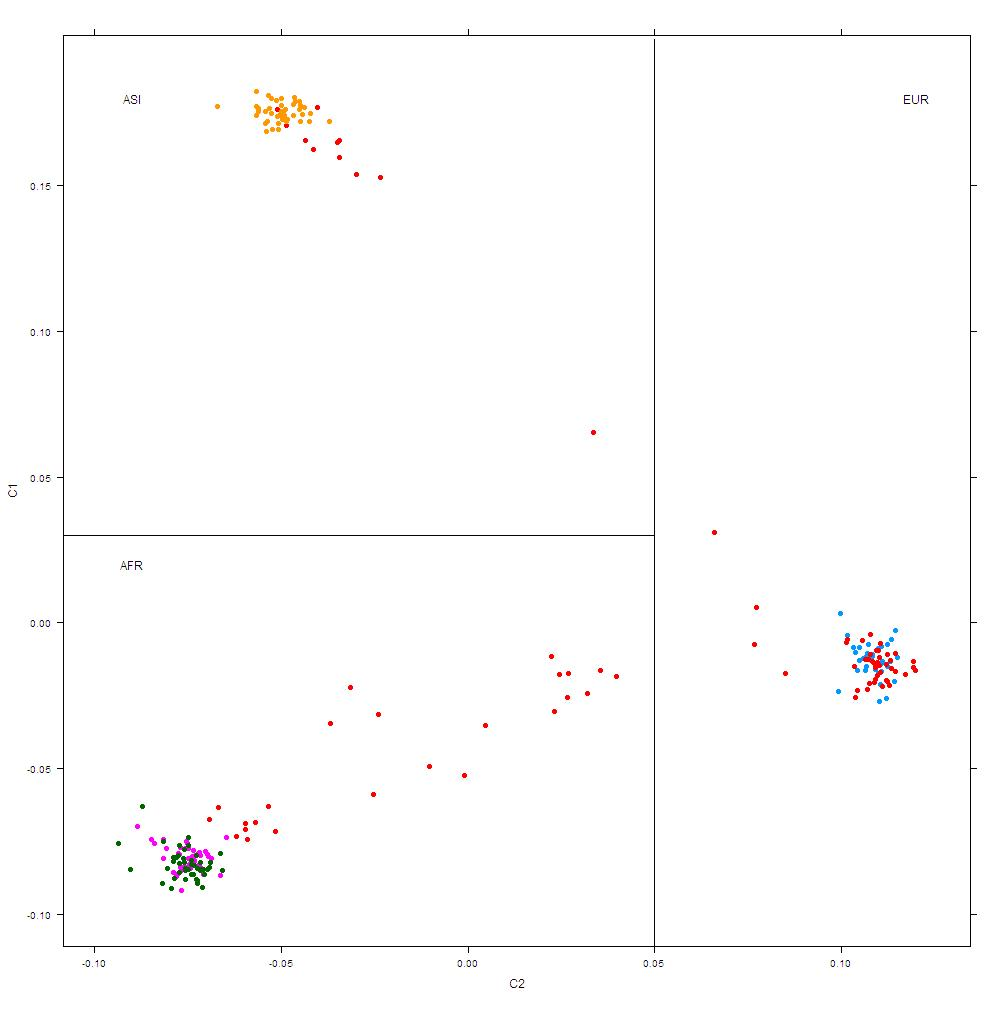


**Figure S1 Classification of patient ethnicity in relation to HapMap populations. Red dots are patients, while blue, yellow, green, and pink dots correspond to European, African, Japanese and Chinese populations, respectively.**

**SNP arrays**

Using SNP array data, we generated both continuous and discrete copy number (CN) values, as well as lesser allele intensity ratio (LAIR) values (ranging from 0 to 1) and balance states, for each of the 81 samples analyzed. The continuous CN values and LAIR values are plotted against each other in figure S2, where color is used to indicate balance state.

**
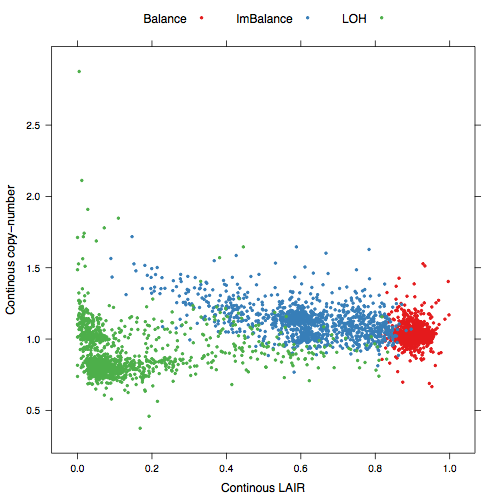
**

Figure S2 Balance state classification in relation to continuous CN and LAIR. Red dots indicate balanced state, blue dots imbalanced state and green dots indicate LOH.

In this plot, balanced segments (showing the same CN for both alleles) are expected to appear on the right side, with a LAIR value near 1. Segments with only one type of allele present (LOH) are expected to appear on the left and show a LAIR value near 0. Segments with different CN for the two alleles (imbalanced) are expected to appear in between these two extremes. Figure S1 shows many segments, classified with LOH, in the intermediate area rather than the left side. This is due to our calling procedure, which categorizes segments not only based on the LAIR values but also takes the discrete CN of the segment into consideration. Such shifts arise when an intermediate LAIR value for a segment with CN 1 is called as LOH.

We also plotted the continuous CN of each segment against its discrete CN (figure S3). The discrete CN assignment to segments is done in such a way that the average discrete CN values across all segments should be close to the sample DNA index (DI). As can be observed, the continuous and discrete CN show some correlation, but the ranges of continuous CN for the various discrete CN show significant overlap with each other, even after multiplication of the continuous CN by the sample DI. This can be explained by a non-linear relationship between CN and the measured intensity on the array, complicated by the fact that, at least for some segments, there may be some heterogeneity within the sample, i.e. two or more cell populations with different CN. The average CN for such segments will not be an integer number.


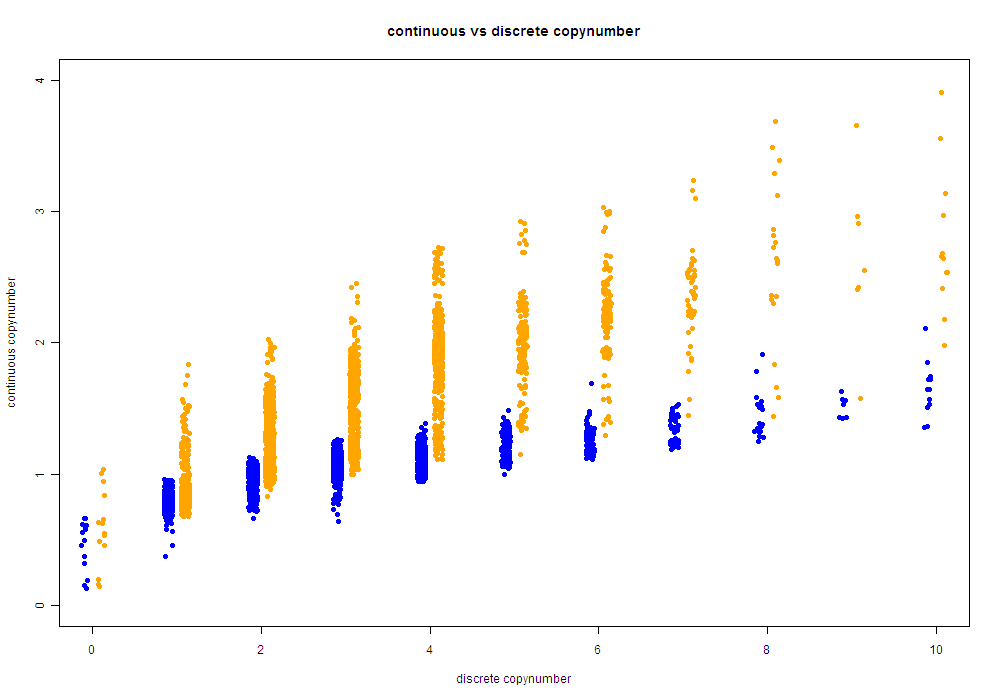


Figure S3 Relationship between discrete and continuous CN. The blue dots represent the continuous CN, while the orange dots represent continuous CN multiplied by sample DI.

**Table S1**

**Clinical details for individual samples**

|  | Age | Ethnicity | DI | Growth pattern | Histological type | Infiltration depth | Lymphnode involvement | Parametrial involvement | Vasoinvasion | Number of breakpoints | Keratin+ populations |
| --- | --- | --- | --- | --- | --- | --- | --- | --- | --- | --- | --- |
| Sample-2 | 52 | AFR | 1.87 | exophytic | squamous | 6-10mm | neg | neg | neg | 20 | 1 |
| Sample-3 | 42 | EUR | 1.08 | exophytic | squamous | >15mm | neg | neg | pos | 51 | 1 |
| Sample-4 | 39 | EUR | 0.94 | exophytic | squamous | 11-15mm | neg | neg | neg | 19 | 1 |
| Sample-5 | 40 | ASI | 1.06 | barrel | squamous | >15mm | pos | neg | neg | 39 | 1 |
| Sample-6 | 67 | ASI | 2.56 | exophytic | ad.squam. | >15mm | pos | neg | pos | 12 | 1 |
| Sample-7 | 64 | EUR | 0.92 | exophytic | ad.squam. | >15mm | neg | neg | pos | 24 | 1 |
| Sample-8 | 40 | EUR | 1.95 | exophytic | ad.squam. | unkown | neg | neg | pos | 20 | 1 |
| Sample-9 | 43 | EUR | 1 | barrel | other | unkown | pos | pos | pos | 32 | 1 |
| Sample-10 | 58 | EUR | 1.97 | barrel | squamous | >15mm | pos | pos | pos | 22 | 1 |
| Sample-11 | 50 | AFR | 1.72 | barrel | squamous | 0.1-5mm | pos | neg | neg | 37 | 1 |
| Sample-12 | 30 | EUR | 1.8 | barrel | ad.squam. | >15mm | neg | neg | neg | 18 | 1 |
| Sample-13 | 71 | EUR | 1.27 | exophytic | ad.squam. | >15mm | neg | neg | pos | 23 | 1 |
| Sample-14 | 33 | EUR | 1.03 | barrel | squamous | >15mm | pos | neg | pos | 34 | 1 |
| Sample-15 | 29 | ASI | 0.97 | exophytic | squamous | >15mm | pos | neg | neg | 13 | 1 |
| Sample-16 | 39 | EUR | 1 | barrel | squamous | >15mm | pos | neg | pos | 14 | 2 |
| Sample-17 | 40 | EUR | 0.96 | exophytic | squamous | >15mm | neg | pos | neg | 32 | 1 |
| Sample-18 | 35 | EUR | 1.07 | barrel | squamous | >15mm | pos | neg | pos | 33 | 1 |
| Sample-19 | 68 | EUR | 0.97 | barrel | squamous | 6-10mm | pos | pos | pos | 2 | 1 |
| Sample-20 | 51 | AFR | 1.88 | barrel | squamous | 6-10mm | neg | neg | neg | 12 | 1 |
| Sample-21 | 28 | AFR | 1.03 | barrel | squamous | 6-10mm | neg | neg | neg | 25 | 1 |
| Sample-22 | 33 | ASI | 0.94 | exophytic | ad.squam. | >15mm | pos | neg | neg | 23 | 1 |
| Sample-25 | 43 | AFR | 1.1 | exophytic | squamous | 6-10mm | neg | neg | neg | 41 | 1 |
| Sample-26 | 55 | EUR | 1.54 | exophytic | squamous | 0.1-5mm | neg | neg | neg | 6 | 1 |
| Sample-27 | 53 | EUR | 1.97 | barrel | squamous | 11-15mm | neg | neg | pos | 26 | 1 |
| Sample-28 | 33 | AFR | 1.47 | barrel | ad.squam. | 11-15mm | pos | pos | pos | 22 | 1 |
| Sample-29 | 44 | AFR | 0.99 | barrel | ad.squam. | 11-15mm | neg | pos | pos | 17 | 1 |
| Sample-30 | 66 | ASI | 1.46 | barrel | ad.squam. | >15mm | pos | neg | unknown | 64 | 1 |
| Sample-31 | 36 | EUR | 1.03 | exophytic | squamous | 6-10mm | pos | pos | pos | 18 | 1 |
| Sample-32 | 43 | AFR | 1.95 | exophytic | squamous | >15mm | neg | neg | neg | 32 | 2 |
| Sample-33 | 66 | ASI | 2.16 | exophytic | squamous | 6-10mm | neg | neg | unknown | 16 | 1 |
| Sample-35 | 61 | AFR | 1.32 | barrel | other | >15mm | pos | neg | pos | 18 | 2 |
| Sample-36 | 47 | ASI | 1.07 | exophytic | squamous | >15mm | pos | neg | pos | 26 | 1 |
| Sample-37 | 31 | EUR | 1.93 | exophytic | squamous | 11-15mm | neg | neg | neg | 27 | 1 |
| Sample-38 | 50 | EUR | 1.1 | exophytic | squamous | unkown | neg | pos | neg | 9 | 1 |
| Sample-39 | 48 | EUR | 1.73 | barrel | ad.squam. | 6-10mm | neg | neg | neg | 33 | 1 |
| Sample-40 | 45 | EUR | 1.95 | exophytic | squamous | >15mm | neg | neg | neg | 9 | 1 |
| Sample-41 | 49 | EUR | 1.21 | barrel | squamous | >15mm | neg | pos | neg | 23 | 1 |
| Sample-42 | 42 | EUR | 0.94 | exophytic | squamous | >15mm | pos | neg | pos | 38 | 2 |
| Sample-43 | 71 | EUR | 1.03 | barrel | squamous | >15mm | pos | neg | pos | 20 | 1 |
| Sample-44 | 49 | EUR | 1.03 | barrel | squamous | >15mm | neg | neg | pos | 35 | 1 |
| Sample-45 | 51 | EUR | 1.24 | exophytic | squamous | 0.1-5mm | neg | neg | neg | 12 | 1 |
| Sample-46 | 53 | EUR | 1.29 | exophytic | squamous | >15mm | pos | pos | neg | 27 | 1 |
| Sample-47 | 53 | AFR | 1.23 | exophytic | squamous | >15mm | neg | neg | neg | 32 | 1 |
| Sample-49 | 57 | EUR | 1.24 | barrel | squamous | 11-15mm | neg | neg | pos | 26 | 1 |
| Sample-50 | 44 | ASI | 0.965 | barrel | squamous | >15mm | neg | pos | pos | 3 | 1 |
| Sample-51 | 35 | EUR | 1.06 | exophytic | adeno | 11-15mm | neg | neg | neg | 9 | 1 |
| Sample-52 | 45 | AFR | 1.8 | barrel | squamous | >15mm | neg | pos | pos | 19 | 1 |
| Sample-53 | 31 | AFR | 1.1 | barrel | ad.squam. | >15mm | pos | neg | neg | 11 | 1 |
| Sample-54 | 30 | AFR | 2.44 | exophytic | squamous | >15mm | neg | neg | neg | 15 | 2 |
| Sample-55 | 40 | AFR | 1.04 | exophytic | ad.squam. | >15mm | neg | neg | neg | 31 | 1 |
| Sample-57 | 73 | EUR | 1.81 | exophytic | squamous | 11-15mm | neg | neg | pos | 16 | 1 |
| Sample-59 | 74 | EUR | 1.62 | exophytic | squamous | 11-15mm | neg | neg | neg | 18 | 1 |
| Sample-60 | 30 | EUR | 1.08 | barrel | squamous | unkown | pos | pos | pos | 33 | 1 |
| Sample-61 | 35 | EUR | 0.99 | barrel | squamous | 6-10mm | neg | neg | neg | 55 | 2 |
| Sample-62 | 41 | EUR | 1.025 | barrel | squamous | >15mm | neg | neg | pos | 38 | 1 |
| Sample-63 | 35 | AFR | 2.07 | barrel | squamous | >15mm | neg | neg | neg | 33 | 1 |
| Sample-64 | 71 | EUR | 1.86 | barrel | squamous | 11-15mm | neg | neg | pos | 20 | 1 |
| Sample-65 | 41 | EUR | 0.94 | exophytic | squamous | 6-10mm | neg | neg | pos | 24 | 1 |
| Sample-66 | 31 | AFR | 1.78 | exophytic | squamous | >15mm | pos | neg | pos | 31 | 1 |
| Sample-67 | 34 | ASI | 1.79 | barrel | squamous | >15mm | neg | pos | neg | 19 | 2 |
| Sample-68 | 32 | AFR | 1.1 | exophytic | ad.squam. | 0.1-5mm | neg | neg | neg | 15 | 1 |
| Sample-69 | 43 | ASI | 1.76 | exophytic | ad.squam. | 6-10mm | neg | pos | neg | 8 | 1 |
| Sample-70 | 33 | AFR | 1.9 | exophytic | squamous | >15mm | pos | neg | neg | 35 | 1 |
| Sample-71 | 56 | AFR | 1.84 | barrel | ad.squam. | >15mm | pos | pos | pos | 44 | 1 |
| Sample-72 | 76 | EUR | 2.05 | barrel | ad.squam. | >15mm | neg | neg | pos | 25 | 1 |
| Sample-73 | 38 | EUR | 0.95 | barrel | squamous | 11-15mm | pos | pos | pos | 22 | 1 |
| Sample-74 | 51 | EUR | 1.04 | barrel | other | >15mm | pos | pos | unknown | 3 | 1 |
| Sample-75 | 62 | EUR | 0.965 | barrel | squamous | unkown | pos | neg | pos | 17 | 1 |
| Sample-76 | 57 | AFR | 1.56 | exophytic | ad.squam. | 11-15mm | neg | neg | neg | 33 | 2 |
| Sample-77 | 66 | EUR | 1.03 | barrel | other | >15mm | pos | neg | unknown | 6 | 1 |
| Sample-78 | 50 | AFR | 1.54 | exophytic | squamous | 11-15mm | pos | neg | neg | 30 | 1 |
| Sample-79 | 44 | AFR | 1.91 | exophytic | adeno | >15mm | neg | neg | pos | 22 | 1 |
| Sample-80 | 54 | EUR | 1.54 | barrel | other | unkown | pos | pos | unknown | 108 | 2 |
| Sample-81 | 45 | EUR | 0.98 | barrel | squamous | >15mm | pos | pos | pos | 28 | 1 |
| Sample-82 | 87 | EUR | 1.03 | barrel | squamous | >15mm | neg | neg | pos | 10 | 1 |
| Sample-83 | 45 | AFR | 0.97 | exophytic | squamous | 11-15mm | neg | neg | neg | 24 | 1 |
| Sample-84 | 25 | EUR | 1.64 | exophytic | ad.squam. | >15mm | neg | pos | neg | 9 | 1 |
| Sample-85 | 42 | ASI | 2.1 | exophytic | ad.squam. | >15mm | pos | pos | pos | 12 | 1 |
| Sample-86 | 37 | EUR | 1.05 | barrel | ad.squam. | >15mm | pos | pos | pos | 13 | 1 |
| Sample-87 | 52 | AFR | 1.08 | barrel | adeno | 11-15mm | pos | pos | pos | 19 | 1 |
| Sample-88 | 40 | AFR | 1.255 | exophytic | other | 11-15mm | neg | neg | unknown | 10 | 1 |

Ethnicity: Classification of ethnicity as determined by PCA, see figure S1

DI: DNA-index, relative amount of DNA in tumor cells compared to unaffected cells

Keratin+ populations: Number of keratin positive populations that could be identified during flow cytometry.

**Table S2: Main characteristics and results of array-CGH studies on cervical tumors** (27-37)

| **Study** | **N** | **FIGO stage** | **Other notes** | **Tumor population** | **Main results** |
| --- | --- | --- | --- | --- | --- |
| Current study | 81 | 1b2 – 2b | Bulky tumors (> 4cm) | Pure tumor cells | Gain in >20% of all samples on 1q, 3q, 5p, 8q, and 20q.  Gain on 3q in > 40%.  Loss in >20% of all samples on 2q, 3p, 4p, 11q, and 13q.  Squamous carcinomas showed significantly more losses on 2q. Adenosquamous carcinomas had more losses on 7p, 7q, and 9p. |
| Hesselmeyer et al. | 30 | 2b - 4 |  | FFPE tumor tissue | Gain on 1q (47%), 3q (77%), 5p (30%), 6p (27%), 20p and 20q (23%).  Loss on 2q (33%), 3p (50%), 4p and q (33%), 8p (23%), and 13q (27%). |
| Dellas et al. | 62 | 1b |  | FFPE tissue containing at least 75% tumor cells | Gain on 3q (15%), 17p (30%), 17q (27%), and 20q (16%).  Loss on 3p (52%), 4p (44%), 4q (53%), 5q (40%), 6q (35%), 13q (45%), and 18q (37%).  Deletions on 9p were more frequent in lymph node positive patients.  Loss on 11p and 18q was related to a worse prognosis in lymph node positive patients.  The clinical parameters invasion depth, vaso-invasion, and histological type were used as confounder but not analyzed separately. |
| Allen et al. | 32 | 1b |  | Microdissection of FFPE tissue | Gain on 3q (44%).  Loss on 3p (56%), 6q (22%), 10q (16%), and 11q (47%).  Total number of gains and losses higher in lymph node positive patients.  No significant differences between lymph node positive and negative patients in chromosome arm analysis. |
| Yang et al. | 20 | 1b (N=16), 2a (3), 2b (1) | Adeno carcinomas, including intestinal and endometrioid type (N=5) | Frozen tumor tissue | Gain on 1q (25%), 1p (30%), 3q (70%), 11q (20%), and 17q (45%).  Loss in 4 of 20 samples on 4q, 13q, or 18q. |
| Umayahara et al. | 18 | 1a1-1b1 | Squamous tumors | FFPE tissue containing tumor | Gain on 1p (33%), 1q (42%), and 3q (67%).  Loss on 2q (33%), 3p (25%), 4p (25%), 4q (25%), 6p (25%), 11p (25%), 11q (67%), and 17p (25%). |
| Rao et al. | 77 | 1b – 4b | Adeno (N=5) and squamous (72) | Biopsies containing > 60% tumor cells | Gain on 1p (27%), 1q (20%), 3q (55%), 5p (30%), 8q (26%), 9q (25%), 13q (16%), 19p (17%), 20p (18%), and 20q (26%).  Loss on 2q (57%), 3p (29%), 4p (30%), 4q (36%), 11q (36%), 13q (43%), and 17p (30%).  No correlation with tumor diameter or histological type. |
| Hidalgo et al. | 10 | unknown |  | Biopsies containing > 70% tumor cells | Gain on 3q (59%), 5p (53%), 7p (53%), and 11p (47%).  Loss in 47% of the tumors on 3p and in >29% on 1p, 4q, 8p, 9q, 13q, and 18q. |
| Huang et al. | 24 | 1b – 2b |  |  | Gain on 1q (37%), 3q (47%), and 8q (20%).  Loss on 2q (20%), 3p (33%), 6q (23%), and 11q (37%).  Higher number of CNA in lymph node positive patients. Separate analysis of gains and losses showed no significant differences.  More frequent gain on 3q and loss on 11q in lymph node positive patients. |
| Wilting et al. | 16 | 1b – 3b and unknown (N=3) | Adeno (N=7) and squamous (9) | Frozen tissue containing > 70% tumor cells | CNA that were found in > 25% of all samples are reported:  Gain on 1q (69%), 3q (69%), and 20q (63%).  Loss on 8p (31%), 10q (31%), 11q (44%), and 13q (50% on one location and 38% on another location).  Higher numbers of gains in squamous tumors, predominantly gain at 3q. |
| Lyng et al. | 20 | 2b – 4b | Squamous (N=19) and adenosquamous (N=1)  Study on intra-tumor heterogeneity | 55 biopsies of 20 tumors | The most frequent gains were found on 1q, 3q, 5p, 8q, 19q, 20q and 22q (>50%).  The most frequent loss on 4p (50%) and 13q (30%).  The most frequent homogeneous aberrations:  Gain of 3q (65%), 20q (65%) and 5p (50%). |
| Lando et al. | 102 | 1b (N=6), 2 (57), 3 (35) 4a (4) | Squamous (N=96), Adeno (1) and Adenosquamous (5) |  | The most common alterations were:  Gain on 1q, 3q, 5p, 20q, and Xq.  Loss on 2q, 3p, 4p, 11q, and 13q.  Clinical parameters were analysed in relation to outcome after chemoradiotherapy. |
